# Effective Surface Treatment for High-Performance Inverted CsPbI_2_Br Perovskite Solar Cells with Efficiency of 15.92%

**DOI:** 10.1007/s40820-020-00509-y

**Published:** 2020-08-19

**Authors:** Sheng Fu, Xiaodong Li, Li Wan, Wenxiao Zhang, Weijie Song, Junfeng Fang

**Affiliations:** 1 Ningbo Institute of Materials Technology and Engineering, Chinese Academy of Sciences, Ningbo, 315201 People’s Republic of China; 2 Center of Materials Science and Optoelectronics Engineering, University of Chinese Academy of Sciences, Beijing, 100049 People’s Republic of China; 3 School of Physics and Electronics Science, Engineering Research Center of Nanophotonics & Advanced Instrument, Ministry of Education, East China Normal University, Shanghai, 200241 People’s Republic of China

**Keywords:** CsPbI_2_Br, Inverted perovskite solar cells, Effective passivation, *V*_*oc*_ loss, Stability

## Abstract

**Highlights:**

- A simple and multifunctional surface treatment strategy is proposed to address the inferior-performance inverted CsPbI_2_Br perovskite solar cells (PSCs).
- The induced-ions exchange can align energy levels, passivate both GBs and surface, and gift the solid protection from external erosions.
- The inverted CsPbI_2_Br PSCs reveal a champion efficiency of 15.92% and superior stability after moisture, operational, and thermal ages.

**Abstract:**

Developing high-efficiency and stable inverted CsPbI_2_Br perovskite solar cells is vitally urgent for their unique advantages of removing adverse dopants and compatible process with tandem cells in comparison with the regular. However, relatively low opening circuit voltage (*V*_*oc*_) and limited moisture stability have lagged their progress far from the regular. Here, we propose an effective surface treatment strategy with high-temperature FABr treatment to address these issues. The induced ions exchange can not only adjust energy level, but also gift effective passivation. Meanwhile, the gradient distribution of FA^+^ can accelerate the carriers transport to further suppress bulk recombination. Besides, the Br-rich surface and FA^+^ substitution can isolate moisture erosions. As a result, the optimized devices show champion efficiency of 15.92% with *V*_*oc*_ of 1.223 V. In addition, the tolerance of humidity and operation get significant promotion: maintaining 91.7% efficiency after aged at RH 20% ambient condition for 1300 h and 81.8% via maximum power point tracking at 45 °C for 500 h in N_2_. Furthermore, the unpackaged devices realize the rare reported air operational stability and, respectively, remain almost efficiency (98.9%) after operated under RH 35% for 600 min and 91.2% under RH 50% for 300 min.

**Graphic Abstract:**

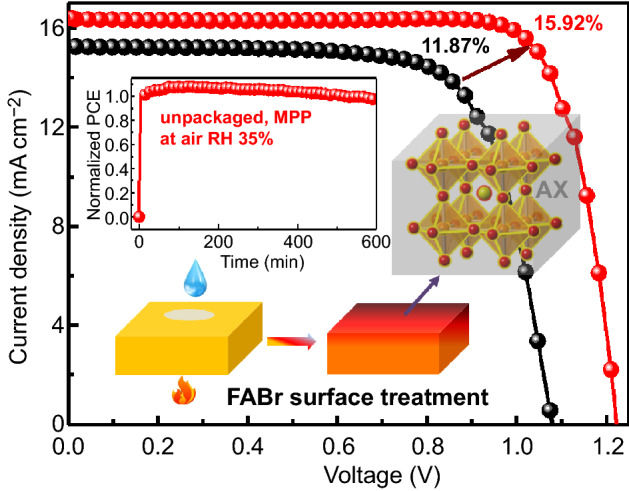

**Electronic supplementary material:**

The online version of this article (10.1007/s40820-020-00509-y) contains supplementary material, which is available to authorized users.

## Introduction

Cesium lead halide (CsPbX_3_) inorganic perovskite materials have attracted great attention recent years, attributed to their excellent thermal stability and superior photoelectric properties [1–6]. Among them, endowed with good phase stability and appropriate light absorption (1.81–1.92 eV), CsPbI_2_Br reveals the most promising for photovoltaic application like top cell of tandem cells [7, 8]. To date, a high efficiency of 16.79% has been realized with a regular structure (n-i-p) CsPbI_2_Br perovskite solar cells (PSCs) [9]. However, as universally used HTL materials, the 2,2′,7,7′-tetrakis (N,N-dipmethoxyphenylamine)-9,9′-spirobifluorene (Spiro-OMeTAD) and poly[bis(4-phenyl) (2,4,6-trimethylphenyl)amine] (PTAA) always need additional dopant of hygroscopic bis(trifluoromethanesulfonyl)imide lithium salt (Li-TFSI) and low-boil-point tert-butylpyridine (t-BP) to increase holes mobility [10–14]. The undesired dopants, which would open channels for moisture erosions and suffer from the uneven distribution under evaluated temperature, gift devices poor moisture/thermal stability and seriously limit their long-term used under real working condition [15, 16]. In contrast, the inverted PSCs (p-i-n) are not necessary to use adverse dopants. Besides, the inverted devices feature the compatible with potential application of tandem solar cells for CsPbI_2_Br [17–19]. Nevertheless, progress of inverted CsPbI_2_Br PSCs has been lagged far from the regular and presents relative low efficiency around 15% [20–22]. In comparison, the major efficiency limitation of the inverted devices is mainly attributed to the larger opening circuit voltage (*V*_*oc*_) loss.

One origin for *V*_*oc*_ loss is the energy level mismatch between charge-transporting layers (CTLs) and CsPbI_2_Br, which would influence carrier transport and cause interfacial energy loss [23–25]. At present, researches have proposed interface engineering with well-matched CTLs to ameliorate interfacial energy loss and obtained considerable ameliorations [26, 27]. Apart from the interfacial energy loss, massive dangling bonds (defects) distributing at surface and grain boundaries (GBs) in the polycrystalline CsPbI_2_Br films would also result in *V*_*oc*_ loss for non-radiative recombination [28]. Besides, the dandling bonds are energetically instable and lead to the phase transition or phase separation for CsPbI_2_Br films under external erosions, which is the origination for the poor moisture stability and limited operational stability for CsPbI_2_Br [29–31]. Although interface engineering could ameliorate interfacial energy loss, unpacked-surface and grain boundaries (GBs) would impede further improvement of *V*_*oc*_ and long-term stability for inverted CsPbI_2_Br devices. To ameliorate the exposed weakness, ions incorporation in the precursor, such as Ni^2+^ and In^3+^, is employed for crystallization control and GBs passivation in inverted CsPbI_2_Br PSCs, which have shown the great stability promotion [32, 33]. However, the efficiency of related devices is still less than 15%. Thus, proposing an efficient strategy to suppress *V*_*oc*_ loss and package the fragile dangling bonds is very urgent for the progress of inverted CsPb_2_Br PSCs.

In this work, we report a simple method to address the urgent need for inverted CsPbI_2_Br PSCs via effective surface treatment with formamidinium bromine (FABr) under high-temperature annealing. High-temperature annealing can induce ions exchange between FABr and CsPbI_2_Br films and gifts the treated films with several promotions. Firstly, benefiting from the minimized the energy level difference with CTLs, the energy levels of CsPbI_2_Br present the up shift. Secondly, the non-radiative recombination is significantly suppressed for the effective passivation of surface and GBs with FA_x_Cs_1-x_Br_y_I_1-y_ (AX). Moreover, the treatment induces bulk heterojunction to accelerate carrier transport for the gradient distribution of FA^+^ ions in the treated films, which would further reduce the bulk recombination. Besides, Br-rich surface and incorporated FA^+^ can act as the solid moisture barrier. As a result, target devices show a champion efficiency of 15.92% with a *V*_*oc*_ up to 1.223 V, which is comparable with the regular devices (Table S1). Apart from efficiency improvement, the fatal problem of moisture stability is greatly improved and the non-encapsulated devices maintain 91.7% of its opening efficiency after aging at 20% relative humidity (RH) for 1300 h. The treatment also gifts the CsPbI_2_Br PSCs ameliorated thermal and operational stability due to the suppressed phase separation. Furthermore, unpackaged devices reveal outstanding operational stability under ambient conditions: remaining almost initial efficiency (98.9%) after MPP tracking at RH 35% for 600 min (78.2% after 1000 min tracking) and 91.2% after MPP measurement at RH 50% for 300 min. Our work offers a feasible strategy for high-efficiency and stable inverted CsPbI_2_Br perovskite cells.

## Experimental Section

### Materials and Precursor Preparation

All perovskite materials were ordered from Xi’an Polymer Light Technology Corp. The solutions of poly[3-(4-carboxylbutyl)thiophene methylamine (P3CT-N, 1 mg mL^−1^ in methanol) and 6,6-phenyl C61-butyric acid methyl ester (PCBM, 10 mg mL^−1^ in chlorobenzene) were prepared according to the literature [11, 33]. And the perovskite precursor was prepared via dissolving 0.9 M CsI, 0.45 M PbI_2_, and 0.45 M PbBr_2_ in 1 mL co-solvent (DMF:DMSO of 7:3). The solutions were first heated at 100 °C until all materials dissolved and then transferred to 60 °C hotplate for a night. After cooled to room temperature, 20 μL acetic acid was added for crystallization adjustment. The perovskite precursors and P3CT-N solution were filtered with 0.45 µm polytetrafluoroethylene filter before spin-coated.

### Devices Fabrication

The perovskite solar cells were fabricated with a p-i-n structure of ITO/P3CT-N/CsPbI_2_Br/PCBM/C60/2,9-dimethyl-4,7-diphenyl-1,10-phenanthroline (BCP)/Ag. The indium tin oxide (ITO) substrates (2 × 2 cm^2^) were ultrasonically cleaned in detergent, distilled water, acetone, and isopropanol (IPA) for 15 min, respectively. Then the substrates were dried under N_2_ flow and treated with O_2_ plasma for 5 min. And P3CT-N layers were deposited according to the literature [11]. After annealing, the substrates were immediately transferred into glovebox filled with N_2_. The CsPbI_2_Br layers were coated on ITO/P3CT-N substrates by 1200 rpm for 50 s under environment temperature of 29 °C and then annealed at 40 °C for 80 s and 180 °C for 10 min to form α-phase CsPbI_2_Br. The XBr (X are the FA, methylamine: MA, ethylamine: EA and propylamine: PA ions) with various concentrations in IPA were spin-coated on the cold films by 3000 rpm for 30 s. FABr-treated CsPbI_2_Br films were annealed at 150 °C for 8 min, and the other XBr films are annealed at 130 °C for 10 min. After annealing, PCBM layer was coated by 2000 rpm for 30 s. Finally, the substrates were transferred into high vacuum (< 3 × 10^−4^ Pa) for thermal evaporation of 20 nm C60, 8 nm BCP, and 140 nm top electrode Ag with the mask of 0.09 cm^2^ (0.3 × 0.3 cm^2^).

### Characterizations

The *J*-*V* curves were collected in glovebox by a calibrated solar simulator (Newport Inc.) with an AM 1.5G filter and Keithley 2400 source meter under 100 mW cm^−2^. The forward scanning is from − 0.2 V to 1.4 V with scan rate of 0.15 V s^−1^ and 50 ms dwell time for every measuring points. The transmission electron microscope (TEM) samples were prepared by scratching films from substrate and ultrasonic dispersion in chlorobenzene and then dropped the solutions on copper mesh annealed at 120 °C for 10 min. The TEM images were obtained at Talos F200X (ThermoFisher). The EQE spectra were conducted with Newport quantum efficiency measurement system (ORIEL IQE 200TM) equipped with a lock-in amplifier and 150 W xenon lamp. The light intensity of each wavelength was calibrated by the standard Si/Ge solar cell. The crystal structure of various films was identified by the X-ray diffraction (XRD) patterns at the Bruker AXS D8 Advanced (Germany) with Cu Kα radiation ((λ = 0.154 nm). The scanning electron microscopy (SEM) images were collected with the field emission scanning electron microscope (Hitachi, S-4800) at 4 kV, and the EDS analyses were obtained at Verios G4 UC. The photoluminescence spectra and absorption spectra were recorded by Fluorolog-Horiba (excitation wavelength of 400 nm) and Model HP8453, respectively. The lifetime of photo-generated carriers was evaluated by the Delta Flex Fluorescence Lifetime System (Horiba Scientific Com., Japan). The excitation light direction was from perovskite to ITO side. The X-ray photo-electron spectroscopy (XPS) and ultraviolet photoemission spectroscopy (UPS) measurements were conducted with a Kratos AXIS ULTRA DALD and the UPS measured under the He I (21.22 eV) emission line. The electrochemical impedance spectroscopy and C^2^-V spectra were conducted on Chenhua CHI760E electrochemical workstation. The transient photo-current (TPC) decay and transient photo-voltage (TPV) decay were recorded by electrochemical workstation (Zahner, Germany) with 80 mW cm^−2^ white light illuminated. The TOF–SIMS spectra were conducted by TOF–SIMS 5 IONTOF with O_2_ 1 kV.

### Stability Measurements

Thermal stability was estimated by heating the devices at 60 °C hotplate in glove box. The tolerance of moisture for various films and the related devices were compared by aging under the control 40% relative humidity (RH) and 20% RH, respectively. The operational stability of devices was measured by white LED array illuminated with the simulating intensity of 100 mW cm^−2^ (spectra region: 430–800 nm, Suzhou D&R instruments Co., Ltd. PVLT-6001 M-16A) under temperature around 45 °C, and the currents were recorded with time advanced by the Keithley 2400 source meter. The operational stability under air condition was conducted with the calibrated solar simulator (Newport Inc.) under control RH around 35% and 50% at room temperature. All devices for various stability estimations were non-encapsulated.

## Results and Discussion

### Effects on Morphology and Photoelectric Properties

**Fig. 1:**
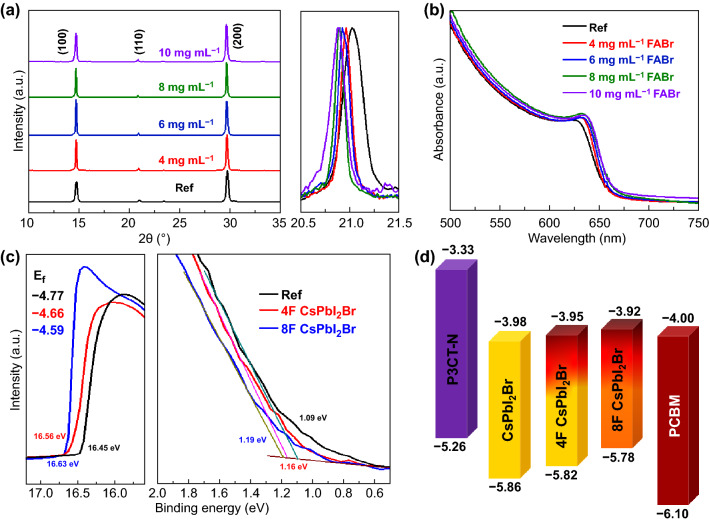
a XRD patterns, **b** UV–Vis absorption spectra, and **c** UPS spectra of the CsPbI_2_Br films treated with various FABr concentrations. **d** Energy level diagram of treated CsPbI_2_Br films and the CTLs

All films reveal the similar XRD patterns of cubic phase as illuminated in Fig. 1a, where the peaks at 14.4°, 21.0°, and 29.4° are corresponding to the (100), (110), and (200) planes [34–36], respectively. When enlarging the (110) planes, the peaks present the gradual shift toward low angle with FABr concentration increase and 10 mg mL^−1^ FABr-treated film reveals the similar diffraction peaks with 8 mg mL^−1^ treated. The high-resolution TEM (Fig. S1) images also reveal enlarged lattice space from 3.10 to 3.18 Å with the Br richer after treatment, which indicates that high-temperature annealing could induce the ions exchange between FABr and CsPbI_2_Br film and partial Cs^+^ ions are substituted by FA^+^ [37]. In parallel, UV–Vis absorption spectra (Fig. 1b) exhibit the consistent redshift absorption (decreased band gap in Fig. S2) for the ions exchange. The treated film shows characteristic signal of N in XPS, corresponding to the existence of FA^+^(compared with Ref in Fig. S3). In addition, the peaks of Cs, I, Br elements reveal shifts after treated, which reveals the changed electron states by the incorporated FA^+^ and ameliorates the moisture tolerance after treatment [33, 38]. Besides, different annealing temperatures for treated films are investigated and the XRD patterns (Fig. S4) present the low-angle shift with the temperature increase, which suggests that the ions exchange needs sufficient potential and 150 °C is suitable. The influence on energy levels of CsPbI_2_Br film after treatment is estimated, and UPS measurements are taken. As shown in Fig. 1c, d, the Fermi energy levels (*E*_*f*_) and valence band maximum (VBM) of treated films both shift toward high energy, which profiles to reducing interfacial energy loss and increasing the build-in electric field of the inverted devices [35, 39]. Thus, high-temperature FABr surface treatment can induce the ions exchange between FABr and CsPbI_2_Br films and suppress interfacial energy loss for higher *V*_*oc*_ in the inverted PSCs.

**Fig. 2:**
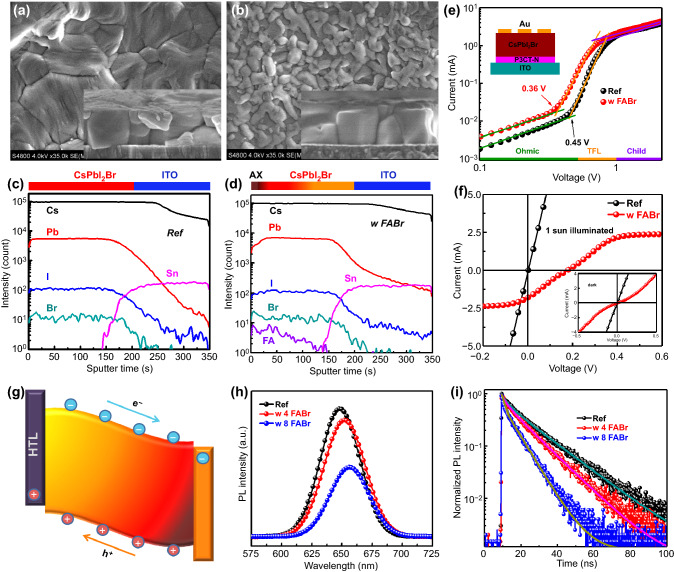
Top view and cross-sectional (inset) SEM images of **a** Ref and **b** 8F CsPbI_2_Br. The TOF–SIMS profiles of **c** Ref and **d** 8F CsPbI_2_Br. **e** Dark I–V plot of the hole-only perovskite device. **f** *I*-*V* curves of the devices with structure of ITO/Au/perovskite/Au measured under dark and 1 sun illuminated. **g** Schematic illustration of the induced gradient band structure promotes the photo-generated carrier transport. **h** PL and **i** TRPL spectra of the Ref film, 4F and 8F films

The morphology changes with various FABr concentration treatment are investigated with SEM (Figs. 2a, b and S5) and atomic force microscope (AFM, Fig. S6). 4 mg/mLFABr-treated film presents the white precipitations around GBs region (Fig. S5b), while the precipitations gradually cover the surface (Fig. S5a-d) with the treated concentrations increasing, and the surface is almost covered when 8 mg mL^−1^ FABr (8F CsPbI_2_Br) was treated (Fig. 2b), which indicates that the ions exchange may proceed from GBs region to surface as the function with treated concentrations. The AFM images reveal the same morphologies as SEM results with surface covered precipitations after treated. In addition, the surface roughness reduces from 30.2 (Ref) to 14.4 nm (8F CsPbI_2_Br), which suggests the treatment could flat the surface and benefit to the contact with PCBM [28, 40]. EDS mappings of Ref and 8F CsPbI_2_Br films are measured to identify the component of the white precipitations. Ref film shows element ratio of consistent stoichiometric ratio of CsPbI_2_Br (Fig. S7) while 8F CsPbI_2_Br film reveals the increased Cs and Br contents while the Pb element shows decrease, which suggests that ions exchange would cover the energetically instable surfaces with the precipitations mainly consisted of FA_x_Cs_1-x_Br_y_I_1-y_ (AX). Meanwhile, the time-of-flight secondary ion mass spectrometry (TOF–SIMS) depth profiling was performed (Fig. 2c, d) to investigate the vertical distribution of elements. Corresponding to the EDS analysis of the AX induced by ions exchange, all elements of the Ref films exhibit the similar distribution while 8F CsPbI_2_Br film reveals the Br-rich and FA-rich surface with reduced Pb and I elements, which would gift CsPbI_2_Br with effective passivation and protections. In addition, FA^+^ ions present a gradient distribution in perovskite films while the other elements display the same distribution with the Ref film, which indicates the GBs are also covered by the AX. Thus, the defects in the treated film would significantly reduce because energetically instable dangling bonds at GBs and surface are both covered with AX after treatment. The space charge limited current (SCLC) examinations with the hole-only device structure are performed, and the trap density could be obtained with filled at the trap-filling limit voltage (*V*_*TFL*_) in Fig. 2e according to the function [41]:

1 $$ n_{t} = 2\varepsilon \varepsilon_{0} V_{TFL} /eL^{2} $$

where *ε* and *ε*_*0*_ are, respectively, the relative permittivity of CsPbI_2_Br (8.5) and the vacuum permittivity, *e* is the electron charge and L is the thickness of perovskite (about 500 nm in Fig. S2f). The calculated trap density shows a significant reduction from 1.69 × 10^14^ to 1.32 × 10^14^ cm^−3^, in good consistence with the effective passivation of AX.

According to the literature [42, 43], the gradient distribution of FA^+^ ions may induce a vertical bulk heterojunction on treated CsPbI_2_Br films, which would accelerate the carriers transport and further suppress bulk recombination. The vertical bulk heterojunction would induce a build-in electric field, and the films sandwiched between the same electrodes might exhibit a photovoltaic performance. We fabricate the devices with structure of Au/CsPbI_2_Br/Au (15 nm)/ITO to exam the build-in electric field. As shown in Fig. 2f, the device fabricated from the Ref film exhibits ohmic characteristic in both dark and light condition, corresponding to resistance. In sharp contrast, the treated film features non-ohmic *I*-*V* under dark condition and photovoltaic performance with a *V*_*oc*_ of 0.174 V under 1 sun illuminated. Thus, gradient distribution of FA^+^ ions can induce a build-in electric field as shown in Fig. 2g, which might accelerate the photo-generated carriers transport. To investigate the influence of carrier transport dynamic after treatment, the steady-state photoluminescence (PL) spectra and the time-resolved PL (TRPL) spectra are performed. As illuminated in Fig. 2h, the PL peaks of the treated films reveal redshift, which consists with the FA^+^ substitution into the CsPbI_2_Br lattice. In addition, the treated films present the quenched PL intensity. According to the literature [28, 44], the PL process in perovskite is attributed to the trap-assisted recombination around GBs and the radiative recombination in grains. The induced AX can effectively passivate the defects to suppress non-radiative recombination. Thus, the quenched PL intensity would be caused by the accelerated carriers transport for the gradient distribution of FABr. The TRPL curves (Fig. 2i) reveal the consistent result of the shortest lifetime 7.15 ns (the fitting date are listed in Table S2) for 8F CsPbI_2_Br, indicating that this treatment can induce a heterojunction to accelerate the carriers transport and further suppress the bulk recombination.

**Fig. 3:**
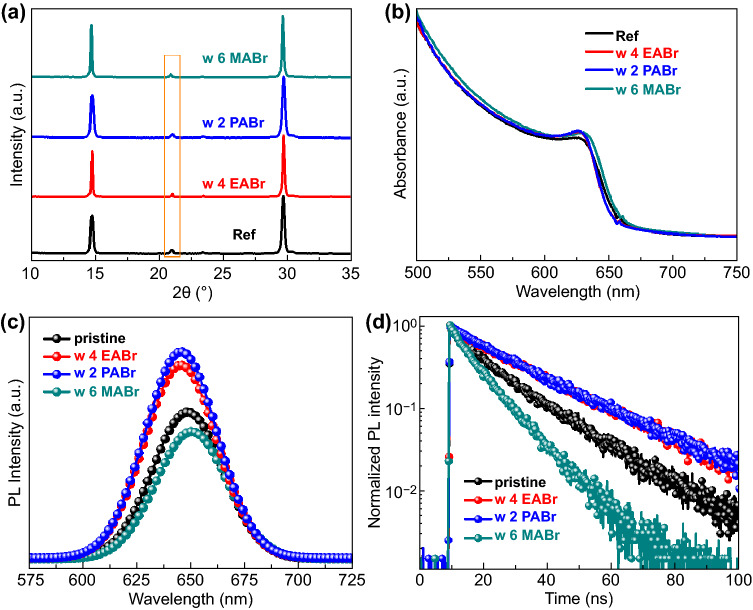
**a** XRD pattern, **b** UV–Vis absorption spectra, **c** PL spectra, and **d** TRPL spectra of Ref, 4 mg mL^−1^ EABr, 2 mg mL^−1^ PABr and 6 mg mL^−1^ MABr-treated films

MABr, EABr, and PABr are taken with high-temperature annealing treatment. As illuminated in Fig. 3a, all XRD patterns show the α phase diffraction peaks and the (110) peaks of the EABr- and PABr-treated CsPbI_2_Br films present no shift while a low-angle shift of MABr-treated films, which may suggest that EA^+^ and PA^+^ ions only terminate on surface and MA^+^ could substitute Cs^+^ in CsPbI_2_Br lattice. The UV–Vis absorption spectra (Fig. 3b) well consist to the XRD results, and there is the blueshift absorption for the EABr and PABr treatment while redshift for MABr treatment, which indicates the MABr treatment may realize the similar promotion with FABr treatment. To observe the morphology change, SEM images of various films are conducted (Fig. S9). EABr- and PABr-treated films exhibit the changed morphology without precipitation after high-temperature annealing, which might be attributed to the secondary grains growth while tuning the surface [36, 42]. In comparison, MABr-treated film displays the same phenomena to FABr treatment with white precipitations on the surface. Additionally, the EABr- and PABr-treated films reveal blueshifted PL peaks with enhanced intensity while the MABr-treated films present the quenched PL emission (Fig. 3c). And the TRPL spectra (Fig. 3d) also show the consistent results with the prolonged lifetime for the EABr- and PABr-treated films and quenched lifetime for MABr-treated film shows. Thus, it could conclude that large ion radius (EA^+^ and PA^+^) could only terminate the surface to suppress surface recombination even via high-temperature annealing. In contrast, FA^+^ and MA^+^ ions could diffuse into CsPbI_2_Br lattices with a heterojunction and effective passivation for high-performance inverted CsPbI_2_Br PSCs after high-temperature annealing.

### Photovoltaic Device Performance

**Fig. 4:**
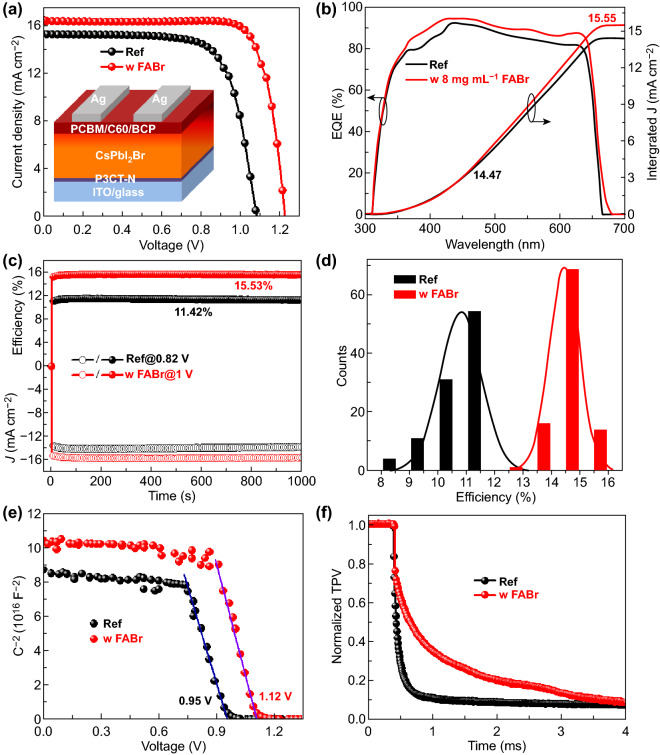
**a** *J*-*V* curves of the champion devices fabricated from the 8F film (red line) and Ref (black line), and the inset image is the schematic of device. **b** EQE spectrum of related devices. **c** Stabilized maximum power out with the champion devices. **d** Efficiency statistic of the Ref and 8F CsPbI_2_Br cells. **e** Mott–Schottky spectra and **f** TPV analysis of the Ref and 8F devices

To estimate the effectiveness on photovoltaic performance with the treatment, inverted CsPbI_2_br PSCs as shown in Fig. 4a inset (cross-sectional SEM images in Fig. S10a) are fabricated. The photovoltaic parameters of devices with various FABr concentration are listed in Table 1. The Ref devices show inferior photovoltaic performance with champion efficiency of 11.87%, *V*_*oc*_ of 1.079 V, short-circuit current density (*J*_*sc*_) of 15.24 mA cm^−2^ and FF of 72.13% (Fig. 4a). In comparison, devices fabricated from the treated CsPbI_2_Br films exhibit a remark enhanced *V*_*oc*_ and fill factor (FF), attributed to the effective passivation and well-matched energy levels. The optimized treated concentration of FABr is 8 mg mL^−1^ and presents the champion efficiency of 15.92% with a *V*_*oc*_ of 1.223 V and a FF of 79.62%, which is the highest efficiency of inverted CsPbI_2_Br-based solar cells and comparable to the regular ones (Table S1). Also, the hysteresis phenomena are suppressed after FABr treatment and the reversed scanning efficiency (Fig. S10b and Table S3), which might benefit from the efficient passivation and adjusted energy levels. The devices prepared from MABr-, EABr-, and PABr-treated films also present ameliorated efficiency and suppressed *V*_*oc*_ loss, and the MABr treated shows the highest efficiency of 13.78% (Fig. S11 and Table S4). The external quantum efficiency (EQE) spectrum of 8F CsPbI_2_Br device reveals a redshift absorption edge and the promoted absorption at long wavelength region compared with Ref in Fig. 4b, which correspond to the incorporation of FA^+^ ions and reduced trap density. The 8F CsPbI_2_Br device shows an integrated photocurrent density of 15.55 versus 14.47 mA cm^−2^ for Ref, in good agreement with the measured *J*_*sc*_ from *J*-*V* curves. In addition, the FABr-treated device reveals a stable output efficiency of 15.53% under the 1 V bias, while Ref device shows an efficiency of 11.42%. Reproducibility is a key issue, and 100 individual devices of each condition are taken into statistics. As presented in Figs. 4d and S12, FABr-treated devices show great improvement of *V*_*oc*_ and FF comparing to Ref, which also suggest the suppression of non-radiative recombination via FABr treatment. Thus effective passivation and induced gradient band structure give the treated devices higher statistics efficiency (14.65 ± 0.55%) than Ref (10.82 ± 0.75%).

**Table 1:** The photovoltaic parameters and average efficiency of various FABr concentration treated devices

Concentration (mg mL^−1^)	*V*_*oc*_ (V)	*J*_*sc*_ (mA cm^−2^)	FF (%)	PCE (%)
Ref	1.079	15.24	72.13	11.87 (10.82 ± 0.75)
4	1.157	16.06	77.21	14.35 (13.65 ± 0.72)
6	1.212	16.42	76.74	15.27 (14.02 ± 0.74)
8	1.223	16.35	79.62	15.92 (14.65 ± 0.55)
10	1.191	15.72	77.77	14.55 (13.42 ± 0.67)

High-temperature FABr surface treatment gifts the CsPbI_2_Br films with well-matched energy levels with CTLs and effective passivation; thus the *V*_*oc*_ loss is significantly suppressed. To estimate the *V*_*oc*_ loss, the capacitance–voltage (*C*-*V*) characterizations is also conducted. Figure 4e shows the *C*^−*2*^-*V* plots of two kinds devices and built-in potential (*V*_*bi*_) are fitted based on Mott–Schottky equation [45, 46]:

2 $$ C^{ - 2} = 2\left( {V_{bi} - V} \right)/(A^{2} \varepsilon \varepsilon_{0} N_{A} ) $$

where *C* is the capacitance under voltage bias (*V*), A is the area of devices, *N*_*A*_ is the carrier concentration, and *ε* and *ε*_*0*_, respectively, present the relative permittivity and vacuum permittivity. The modified device exhibits a large *V*_*bi*_ of 1.12 V than 0.95 V as obtained for the Ref, thus the higher *V*_*oc*_ of the treated devices. The transient photocurrent (TPC) and transient optical-voltage (TPV) decay are also measured to study the change of charge-transport properties after FABr treated. The TPC spectra (Fig. S13a) present a minimized response for treated devices, which indicates the faster of carrier extraction and transmission due to the suppressed defects and induced gradient band structure [45, 47]. In parallel, the TPV measurements (Fig. 4f) show a prolonged carriers lifetime for the treated device, corresponding to the minimized trap density [48]. To further insight the charge-carrier recombination for the related devices, the electrochemical impedance spectrum (EIS) is performed (Fig. S13b) and the FABr-treated device displays a larger *R*_rec_ than the Ref, contributing from the effective passivation and accelerated carriers transport for the treated films [11, 28].

### Devices Stability

**Fig. 5:**
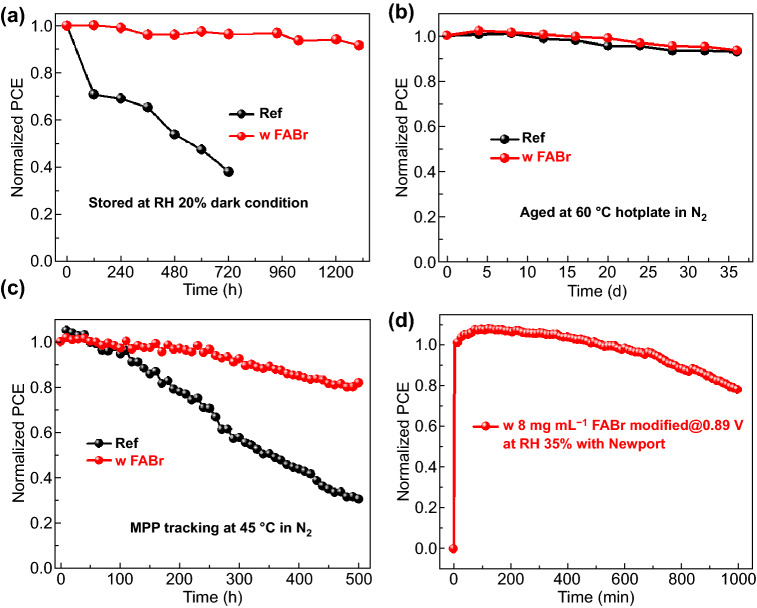
**a** Moisture stability of the related devices measured under controlled RH 20% ambient condition. **b** Thermal stability of the corresponding devices aged at 60 °C hotplate. **c** MPP tracking of various devices at 100 mW cm^−2^ illuminated at 45 °C. **d** MPP measurement with Newport under controlled RH 35% ambient condition

Apart from efficiency limitation, phase transition under moisture condition is another lethal point for CsPbI_2_Br solar cells [49, 50]. As shown in Fig. S14a, the treated films show delayed phase transition compared with the Ref film and the FABr-/MABr-treated films show slower. After 4 h aging, the FABr-treated films exhibit the unchanged images and absorption property (Fig. S14) while the other films have presented significant phase transition, proving that FABr is more compatible to protect CsPbI_2_Br films. In comparison with the Ref, FABr-treated film remains α phase, while the Ref has totally transited into γ phase after 5 h aging (Fig. S15), which indicates this treatment gifts the related devices with relative long-term moisture stability. The moisture stability of the devices is investigated by aging under control RH 20% ambient condition. As shown in Fig. 5a (*J*-*V* curves shown in Fig. S16), the FABr-treated device exhibits good moisture stability and maintains 91.7% of its initial efficiency after 1300 h aging, while the Ref has suffered from significant attenuation with 38.3% at 720 h, derived from the solid humidity shield of AX, Br-rich surface and FA^+^ incorporation.

To investigate whether the FABr treatment ruins the thermal stability, we store the devices at 60 °C in glovebox and measure the efficiency at regular time. As presented in Fig. 5b, the treated device and Ref reveal the similar trend and remain 93.6% efficiency after aging for 36 d (*J*-*V* curves in Fig. S17), which indicates this treatment also features good thermal stability. As the key issue of solar cells, operational stability of related devices is taken into comparison via MPP tracking under 100 mW cm^−2^ white LED array illuminated at 45 °C (Fig. 5c). The FABr-treated device displays the better operational stability and remains 81.8% of its opening efficiency after MPP tracking for 500 h while Ref has significantly decreased (the other parameters are presented in Fig. S18). According to previous studies [51–53], the limited operational stability of CsPb(IBr)_3_-based cells is mainly attributed to the phase segregation around GBs. Attributed to the effective passivation by the induced AX and Br-rich for the treated films, the phase segregation gets effective suppression (Fig. S19) and thus endows treated device with decreased hysteresis and promoted operational stability. Associating the good moisture stability and promoted operational stability for the treated devices, we further conduct the MPP measurement under RH 35% ambient condition with Newport. As shown in Fig. 5d (*J*-*V* curves in Fig. S20b), the non-encapsulated device remains 98.9% of its initial efficiency after 600 min tracking and 78.2% after 1000 min measuring. Moreover, the operational stability is further evaluated under high humidity (RH 50%) as shown in Fig. S20a-c. After MPP measurement at 0.9 V bias for 300 min, the device remains 91.2%.

## Conclusion

In summary, we have developed an effective strategy for high-efficiency and stable inverted CsPbI_2_Br perovskite solar cells via high-temperature FABr surface treatment. The *V*_*oc*_ loss is significantly suppressed benefiting from the adjusted energy levels and effective passivation with promoted carrier transport. Furthermore, the FA^+^ substitution of CsPbI_2_Br lattice and Br-rich surface gift the treated films with solid moisture shelter. The resulted devices show great efficiency improvement from 11.87 to 15.92% with promoted *V*_*oc*_ of 1.223 V. Without ruining thermal stability after FABr treatment, the fatal drawback of moisture stability gets significant promotion and the target device remains 91.7% of its initial efficiency after 1300 h comparing with 38.3% at 720 h aging of Ref. Furthermore, the operational stability also reveals an amelioration and the treated device maintains 81.8% of its opening efficiency after MPP tracking at 45 °C for 500 h. More importantly, the treated device without encapsulation remains almost efficiency (98.9%) of its initial efficiency after MPP tracking under RH 35% for 600 min and maintains 91.2% after MPP under RH 50% for 300 min. Our work provides a feasible strategy for high-performance inverted CsPbI_2_Br PSCs.

## Electronic supplementary material

Below is the link to the electronic supplementary material.

Supplementary material 1 (PDF 2539 kb)
