# Supplementary material for: Effective Surface Treatment for High-Performance Inverted CsPbI2Br Perovskite Solar Cells with Efficiency of 15.92%
Source: Nanomicro Lett. 2020 Aug 19;12:170. doi: 10.1007/s40820-020-00509-y (PMC7770732; doi:10.1007/s40820-020-00509-y)
Supplement: Supplementary file 1 — Supplementary material 1 (PDF 2539 kb) [file 40820_2020_509_MOESM1_ESM.pdf]

Supporting Information for

# Effective Surface Treatment for High-Performance Inverted $CsPbI_2Br$ Perovskite Solar Cells with Efficiency of 15.92%

Sheng Fu[1, 2], Xiaodong Li[3], Li Wan[1], Wenxiao Zhang[1, 2], Weijie Song[1, 2], Junfeng Fang[1, 2, 3, *]

[1]Ningbo Institute of Materials Technology and Engineering, Chinese Academy of Sciences, Ningbo 315201, People's Republic of China

[2]Center of Materials Science and Optoelectronics Engineering, University of Chinese Academy of Sciences, Beijing 100049, People's Republic of China

[3]School of Physics and Electronics Science, Engineering Research Center of Nanophotonics & Advanced Instrument, Ministry of Education, East China Normal University, Shanghai 200241, People's Republic of China

*Corresponding author. E-mail: jffang@phy.ecnu.edu.cn (Junfeng Fang)

## Supplementary Table and Figures

**Table S1** A summary of $CsPbI_2Br$-based inorganic perovskite solar cells researches with the detail performance parameters and stability

| Devices structure | $V_{oc}$ (V) | PCE (%) | Stability | Refs. |
|---|---|---|---|---|
| ITO/PEDOT:PSS/$CsPbI_2Br$/PCBM/BCP/Al | 1.06 | 6.8 | non* | [S1] |
| FTO/NiMgLiO/ $CsPbI_2Br$ /PCBM/BCP/Ag | 0.98 | 9.14 | L* 90% for 500 | [S2] |
| ITO/NiOx/$CsPbI_2Br$ /C60/BCP/Ag | 1.05 | 10.4 | non | [S3] |
| FTO/NiO/$CsPbI_2Br$/ZnO/C60/Ag | 1.14 | 13.30 | T* 85% for 360 h | [S4] |
| FTO/NiO/$CsPbI_2Br$:In/ZnO/C60/Ag | 1.15 | 13.74 | M* 100% for 100 h | [S5] |
| FTO/NiMgLiO/$CsPbI_2Br$/$TiO_2$/Ag | 1.26 | 14.00 | T* 92.1% for 1000 h | [S6] |
| ITO/NiOx/$CsPbI_2Br$/$Nb_2O_5$/Ag | 1.20 | 14.11 | S* 98% for 30 d | [S7] |
| FTO/NiMgLiO/$CsPbI_2Br$/$TiO_2$/Sb | 1.28 | 14.8 | L 90% for 1000 h | [S8] |
| ITO/P3CT/$CsPbI_2Br$:Ni/PCBM/C60/BCP/Ag | 1.141 | 13.88 | L 60% for 500 h | [S9] |
| FTO/NiO/$CsPbI_2Br$/ZnO/C60+TPFPB+$LiClO_4$/Ag | 1.23 | 15.19 | S 91.8% for 72 d | [S10] |
| ITO/TPE-S/$CsPbI_2Br$/PCBM/ZnO/Ag | 1.26 | 15.4 | L 84% for 400 h | [S11] |
| ITO/P3CT-N/$CsPbI_2Br$:FABr /PCBM/C60/BCP/Ag | 1.223 | 15.92 | M 91.7% for 1300 h; L 81.8% for 500 h; T 93.6% for 36 d | Our |

| | | | | |
|---|---|---|---|---|
| ITO/$SnO_2$/$CsPbI_2Br$/Spiro/Au | 1.22 | 14.21 | non | [S12] |
| FTO/$TiO_2$/$CsPbI_2Br$:Mn/QDs/PTAA/Au | 1.172 | 14.09 | M* 97% for 840 h | [S13] |
| FTO/$TiO_2$/$CsPbI_2Br$:Zn/Spiro/Ag | 1.18 | 13.60 | M 85% for 480 h | [S14] |
| ITO/$TiO_2$/$CsPbI_2Br$:Cu/Spiro/$MoO_3$/Ag | 1.19 | 16.15 | M 95% for 720 h | [S15] |
| FTO/$TiO_2$/$CsPbI_2Br$/QDs/PTAA/Au | 1.22 | 14.81 | M 100% for 720 h | [S16] |
| ITO/$SnO_2$/ZnO/$CsPbI_2Br$/Spiro/$MoO_3$/Ag | 1.23 | 14.6 | non | [S17] |
| ITO/$TiO_2$/$CsPbI_2Br$/Spiro/Au | 1.23 | 16.07 | M 95% for1000 h | [S18] |
| ITO/$SnO_2$/PN4N/$CsPbI_2Br$/PDCBT/$MoO_3$/Ag | 1.30 | 16.2 | L* 90% for 400 h | [S19] |
| FTO/$TiO_2$/$CsPbI_2Br$:Ac/Spiro/Au | 1.30 | 15.56 | M 86% for 336 h | [S20] |
| ITO/$SnO_2$/$CsPbI_2Br$/$CsPbI_xBr_y$/Spiro/Au | 1.32 | 15.50 | T 80% for 350 h | [S21] |
| ITO/$SnO_2$/ZnO/$CsPbI_2Br$/PSQ2/$MoO_3$/Ag | 1.27 | 15.50 | L 83% for 300 h | [S22] |
| ITO/$SnO_2$/$CsPbI_2Br$:CsBr/Spiro/Au | 1.271 | 16.37 | S 86% for 1368 h | [S23] |
| ITO/$TiO_2$/$CsPbI_2Br$/Spiro/Au | 1.12 | 14.69 | M 70% for 20 h | [S24] |
| FTO/$TiO_2$/$CsPbI_2Br$:Eu/Spiro/Au | 1.22 | 13.34 | L 93% for 370 h | [S25] |
| FTO/$TiO_2$/$CsPbI_2Br$ /QDs:FAI/PTAA/Au | 1.223 | 14.12 | M 100% for 30 d | [S26] |
| ITO/ZnO:Cs/$CsPbI_2Br$/Spiro/$MoO_3$/Ag | 1.28 | 16.34 | M 96% for 200 h; T 81% for 200 h | [S27] |
| ITO/$SnO_2$/$CsPbI_2Br$:O/Spiro/Au | 1.18 | 15.17 | M 92.3% for 32 d | [S28] |
| ITO/$TiO_2$/$CsPbI_2Br$ /Spiro/PTAA/Ag | 1.23 | 16.58 | T 90% for 500 h; M 90% for 4000 h; L 90% for 1000 h | [S29] |
| ITO/$SnO_2$/$CsPbI_2Br$/Spiro/Au | 1.286 | 15.58 | M 83.4% for 1540 h; L100% for 350 h | [S30] |
| ITO/$TiO_2$/$CsPbI_2Br$ /Spiro/Au | 1.32 | 16.79 | M 90% for 1000 h; | [S31] |

* T is the thermal stability, M is the moisture stability, S is the storage stability at $N_2$ atmosphere and L is the operational stability. A, A and A present the inverted structure $CsPbI_2Br$ devices, this work and the regular structure $CsPbI_2Br$ devices.

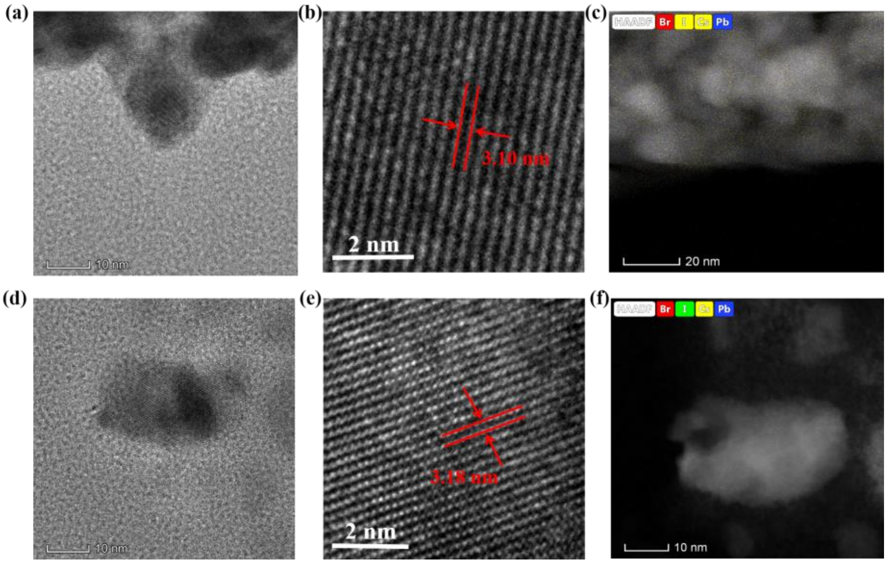


**Fig. S1** TEM images of the Ref and 8F $CsPbI_2Br$ films. (**a-c**) TEM images and EDX

element mapping of Ref. (**b-d**) The TEM images and EDX element mapping of 8F $CsPbI_2Br$. The treated films show an enlarged lattice space, which indicates the incorporation of $FA^+$. From the EDX mapping, the Ref shows a Br/I ratio of 48.58% while treated films present a ratio of 56.43%, which prove the Br-rich.

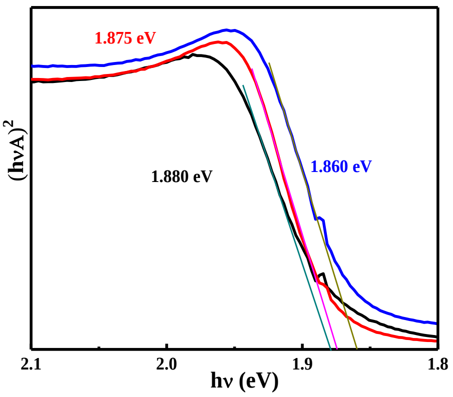


**Fig. S2** Kubelka-Munk spectra of the corresponding films based on the absorption spectrum

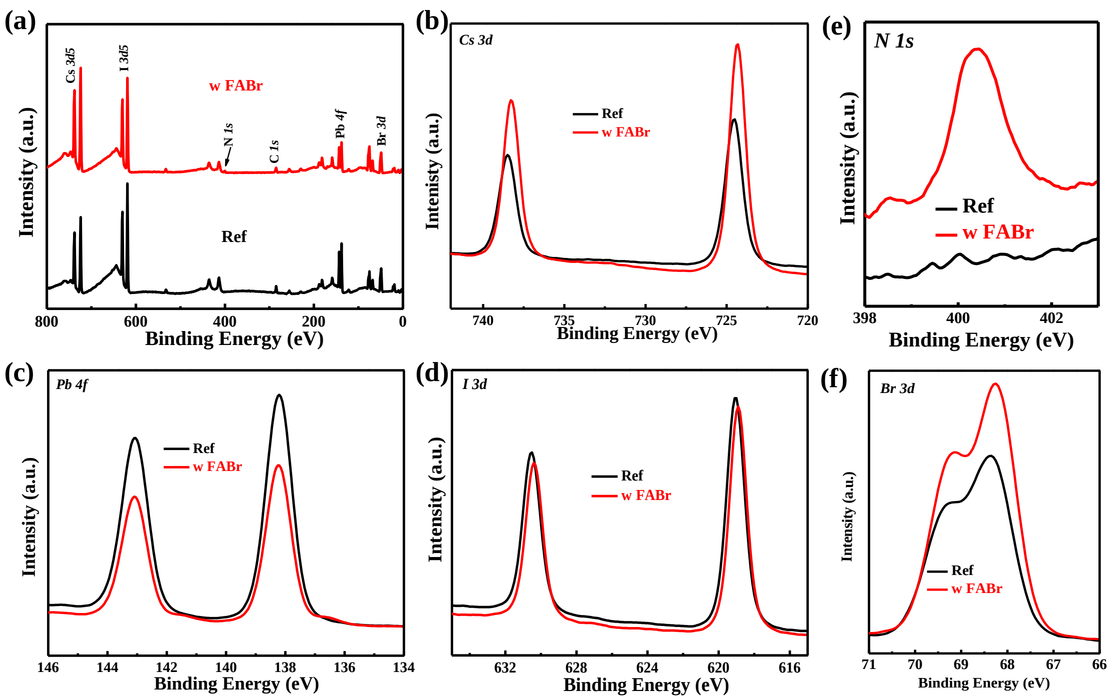


| | Cs | Pb | I | Br | N |
|---|---|---|---|---|---|
| ***Atomic (%)*** | 16.97 | 9.61 | 23.24 | 15.23 | 0.1 |
| ***Atomic (%)*** | 25.6 | 6.65 | 21.62 | 20.06 | 2.19 |

**Fig. S3** The XPS spectrum of Ref and 8F $CsPbI_2Br$ films. (**a**) Full XPS spectra. (**b**) Cs 3d, (**c**) Pb 4f, (**d**) I 3d (**e**) N 1s and (**f**) Br 3d core-level spectra and the quantitative XPS results

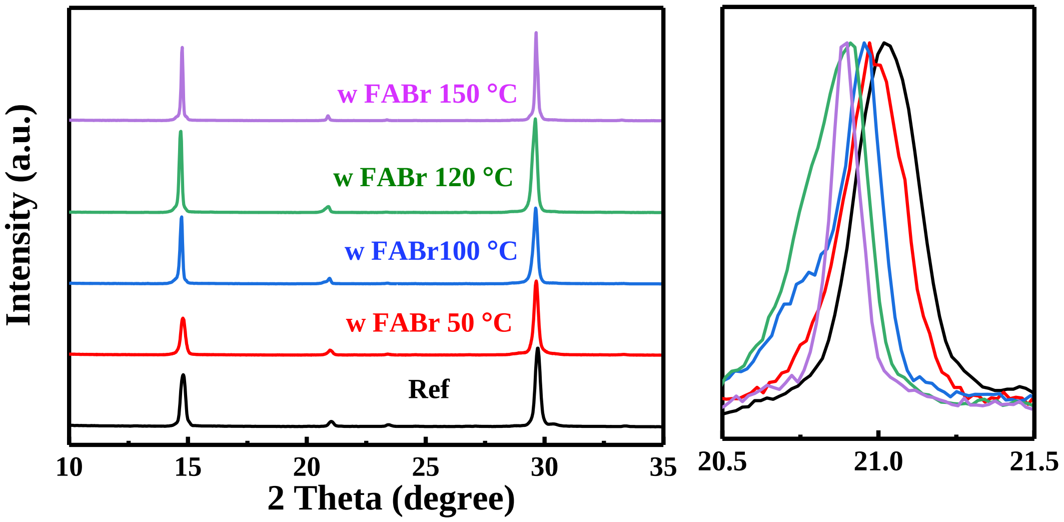


**Fig. S4** XRD patterns of the FABr treating films annealed at various temperature

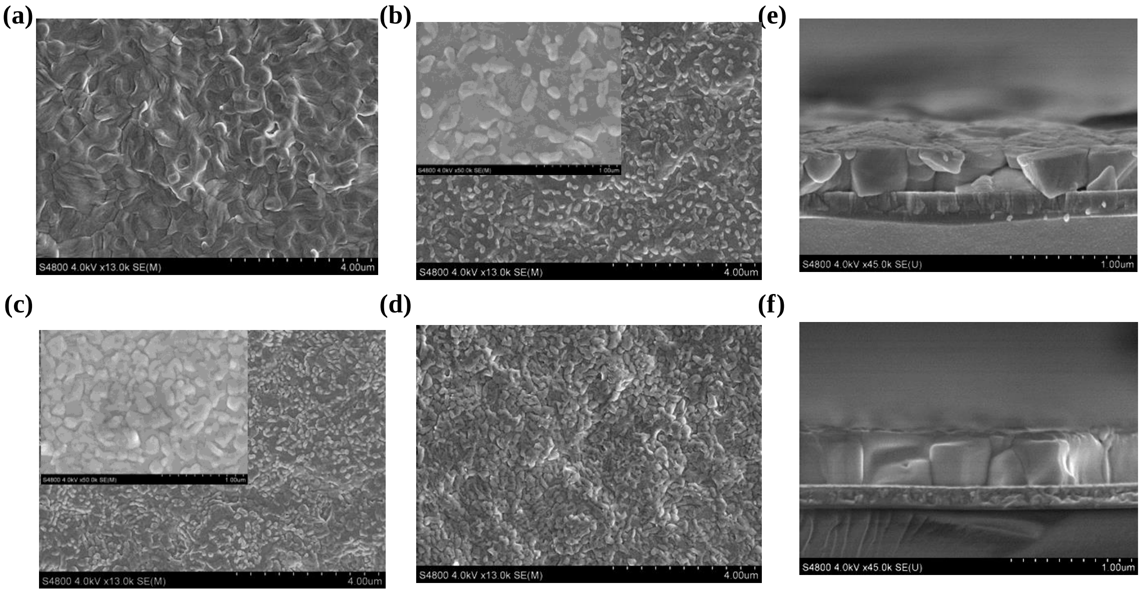


**Fig. S5** Top-view SEM images of (**a**) the Ref, (**b**) 4, (**c**) 6, and (**d**) 8 mg $mL^{-1}$ FABr. The cross-sectional SEM images of (**e**) the Ref and (**f**) the 8 mg $mL^{-1}$ FABr films. With the FABr treated concentration increase, the covered region by AX gradually distribute from the GBs toward full surface and almost covered with 8 mg $mL^{-1}$. Also, the cross-sectional SEM image of treated film reveals a thin consequent covered layer compared with the Ref and the thickness of perovskite is about 500 nm.

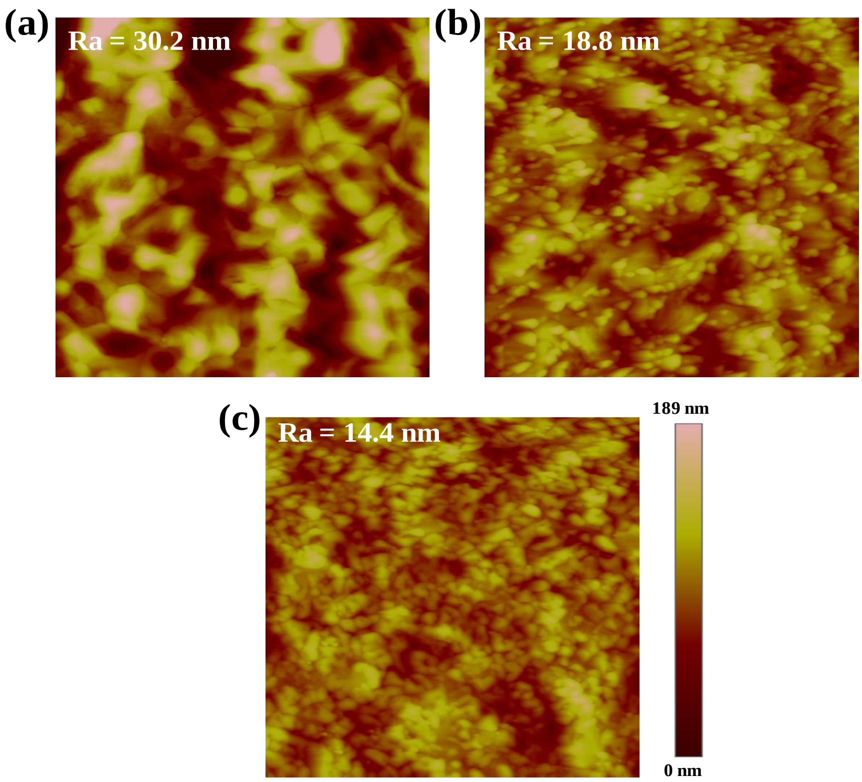


**Fig. S6** AFM images of (**a**) Ref, (**b**) 4F $CsPbI_2Br$ and (**c**) 8F $CsPbI_2Br$ films. The area is 5×5 μm. The modified films show reduced surface roughness, which indicates the better contact with the ETL

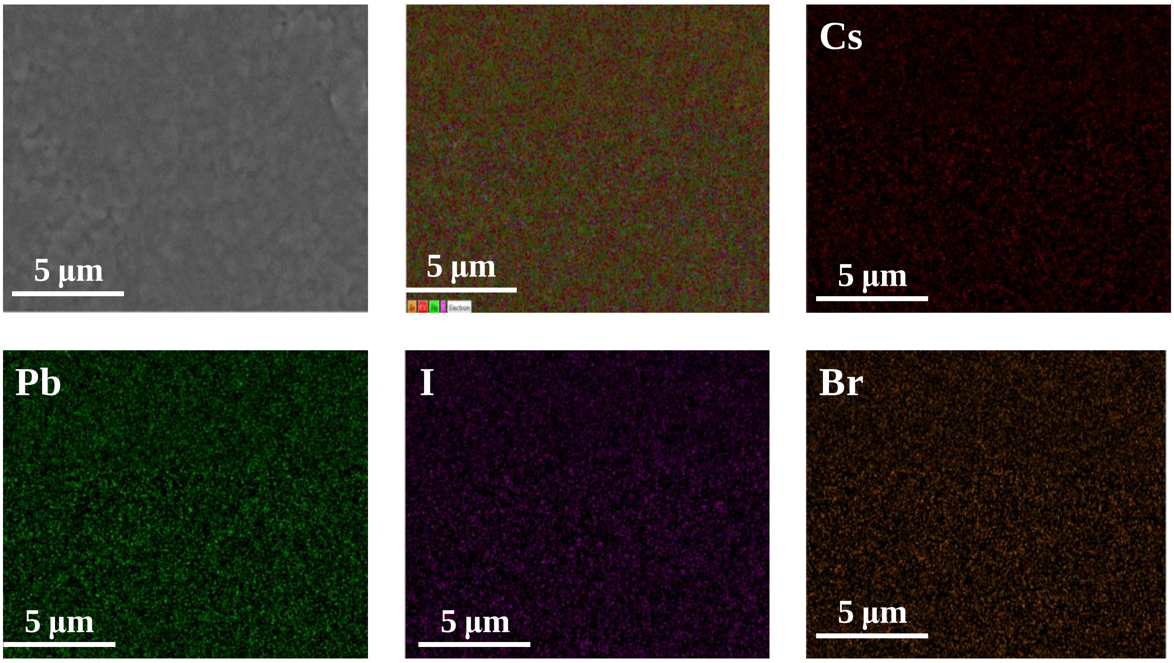


**Fig. S7** The EDX top-view element mapping of Ref film. The element ratio reveals 20.58% of Cs element, 20.12% of Pb element, 40.14% of I element and 19.26% of Br, which consists with the Stoichiometric ratio of $CsPbI_2Br$

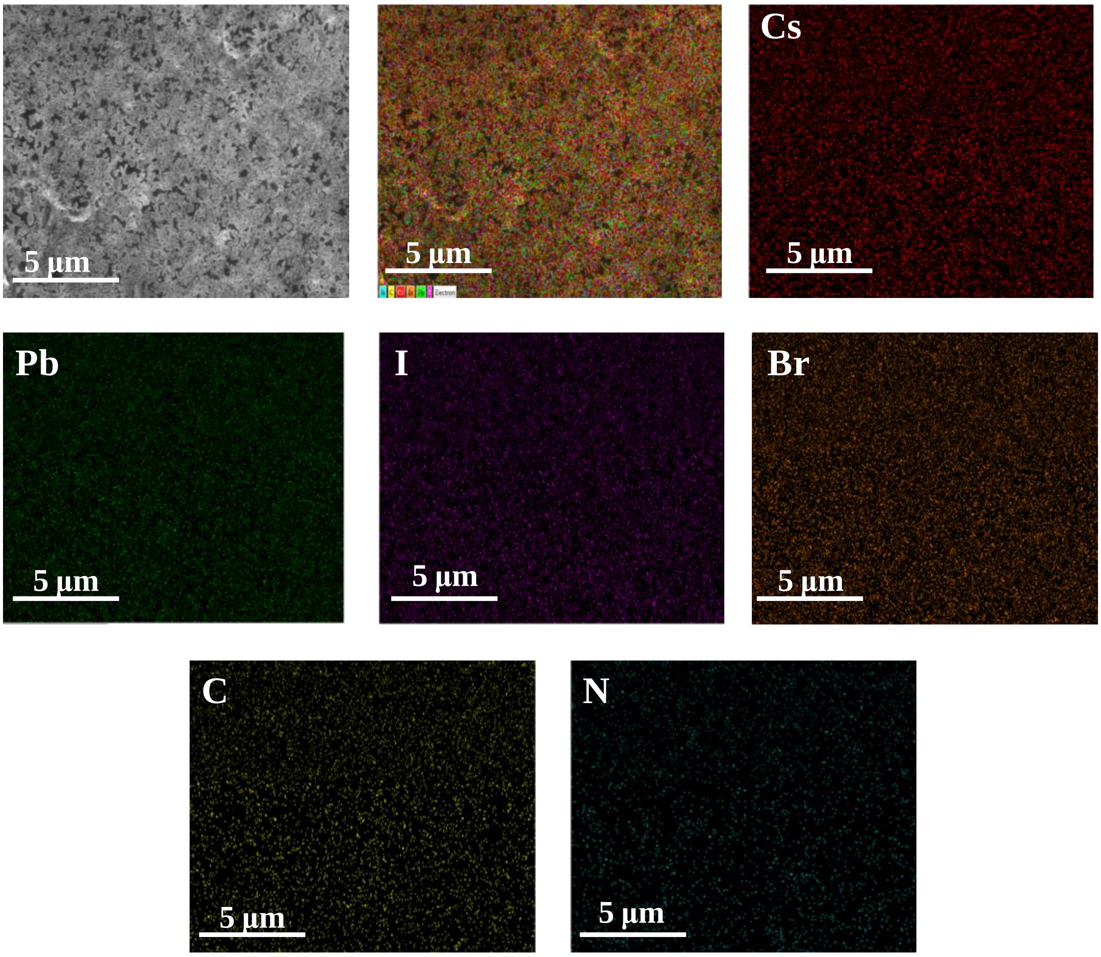


**Fig. S8** EDX top-view element mapping of the 8 mg $mL^{-1}$ FABr treated film. The film reveals the element ratio of 14.75% for C element, 2.08% for N element, 19.40% for Cs element, 14.16% for Pb element, 28.56% for I element and 21.05% for Br element. Associated the changed morphology and the component at surface, it could infer that the covered layer (AX) consists of FA/Cs and Br/I

**Table S2** TRPL fitting results of the various treated films on the glass substrate

| Sample | $\tau_1$ (ns) | $A_1$ | $\tau_2$ (ns) | $A_2$ | $\tau_{avg}$ (ns)* |
|---|---|---|---|---|---|
| Ref | 3.88 | 0.27 | 16.57 | 0.73 | 15.56 |
| 4 FABr | 3.12 | 0.24 | 12.84 | 0.76 | 12.15 |
| 8 FABr | 0.75 | 0.34 | 7.48 | 0.66 | 7.15 |
| 4 EABr | 4.24 | 0.12 | 22.98 | 0.88 | 22.52 |
| 2 PABr | 10.56 | 0.14 | 25.24 | 0.86 | 24.30 |
| 6 MABr | 2.65 | 0.22 | 9.65 | 0.78 | 9.15 |

* The $\tau_{avg}$ is calculated with the formal : $\tau_{avg}= (A_1\tau_1^2+ A_2\tau_2^2)/( A_1\tau_1+ A_2\tau_2)$ [S11].

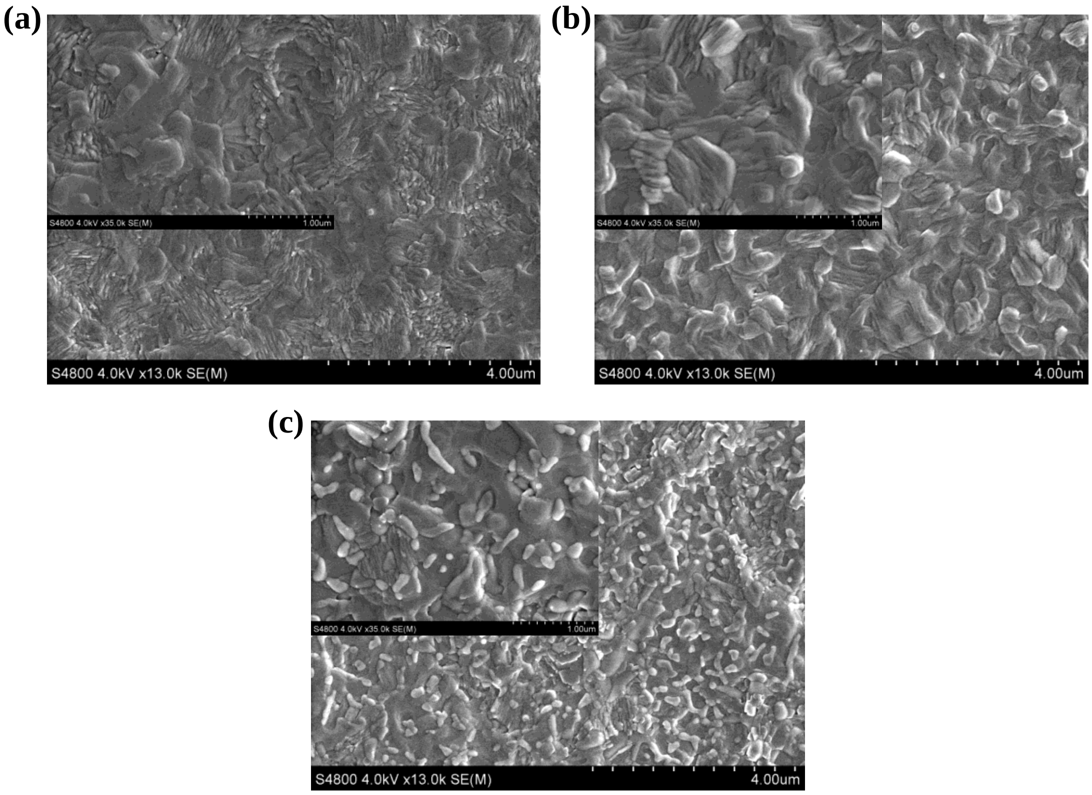


**Fig. S9** Top-view SEM images of (**a**) 4 mg mL$^{-1}$ EABr, (**b**) 2 mg mL$^{-1}$ PABr and (**c**) 6 mg mL$^{-1}$ MABr treated $CsPbI_2Br$ films. The inset images are the enlarged morphology of related films. Comparing to EABr and PABr treatment, MABr treated films exhibits the similar morphology with the second phase covering the surface.

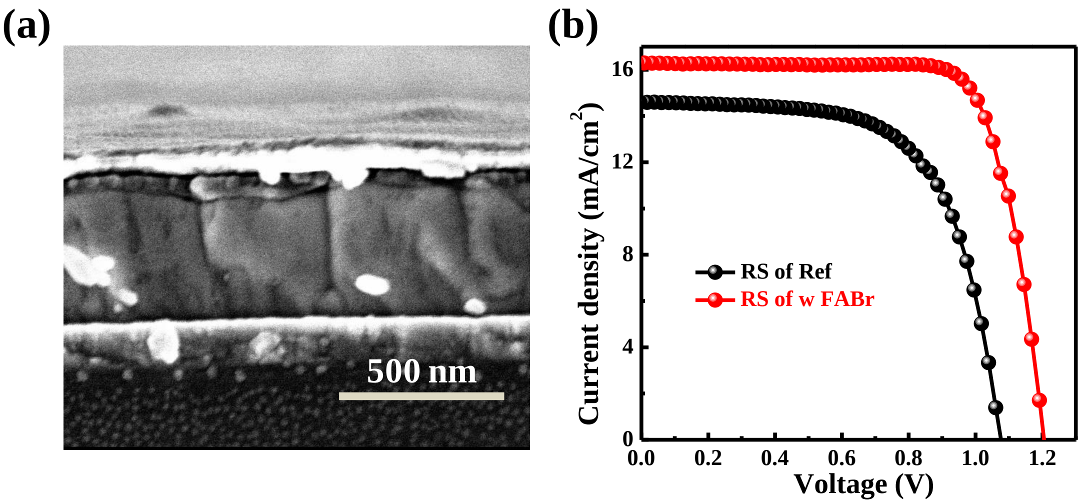


**Fig. S10** (**a**) The cross-sectional SEM image of the inverted $CsPbI_2Br$ device with a structure of ITO/P3CT-N/$CsPbI_2Br$/PCBM/C60/BCP/Ag. The Ag electrode is deposited about 80 nm for convenient sample preparation. (**b**) The reverse scanning *J-V* curves of the champion devices of Ref and 8F treatment

**Table S3** Photovoltaic parameters of the reverse scanning *J*-*V* curves

| Sample | $V_{oc}$ (V) | $J_{sc}$ (Ma cm$^{-2}$) | FF (%) | PCE (%) |
|---|---|---|---|---|
| Ref | 1.075 | 14.60 | 64.26 | 10.08 |
| 8F $CsPbI_2Br$ | 1.201 | 16.03 | 76.24 | 14.97 |

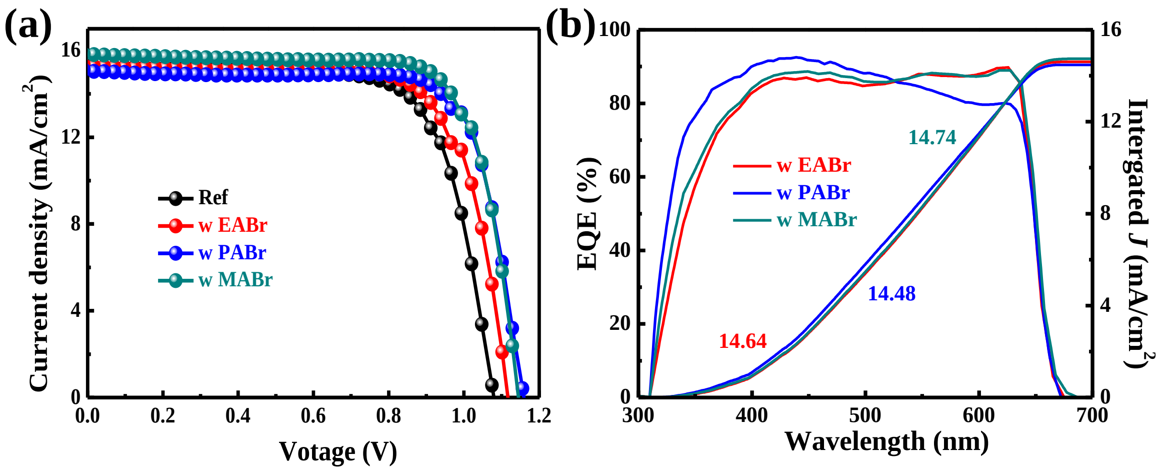


**Fig. S11** J-V curves (**a**) and EQE spectra (**b**) of champion devices with 4 mg mL$^{-1}$ EABr, 2 mg mL$^{-1}$ PABr, and 6 mg mL$^{-1}$ MABr treated

**Table S4** The photovoltaic parameters of the *J*-*V* curves for EABr, PABr and MABr treated devices

| Sample | $V_{oc}$ (V) | $J_{sc}$ (mA cm$^{-2}$) | FF (%) | PCE (%) |
|---|---|---|---|---|
| 4 EABr | 1.117 | 15.26 | 73.46 | 12.53 |
| 2 PABr | 1.159 | 15.04 | 75.16 | 13.10 |
| 6 MABr | 1.145 | 15.80 | 76.15 | 13.776 |

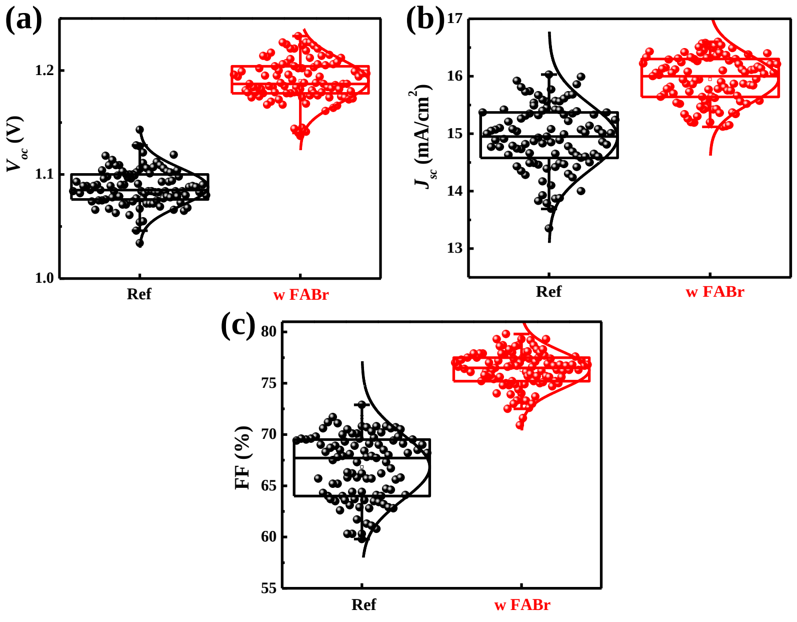


**Fig. S12** Statistic spectrum of the parameter of the Ref and 8F treated devices. Statistics (**a**) Voc, (**b**) Jsc and (**c**) FF from 100 individual devices

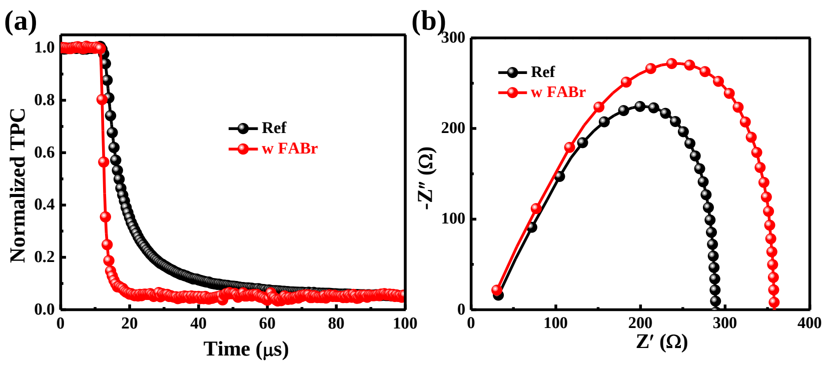


**Fig. S13** (**a**) TPC spectra of the Ref device and 8F treated devices. In comparison with the Ref, the treated device shows a faster carrier transport, which benefits from the suppressed defects and induced gradient band structure. (**b**) Nyquist plots of of the Ref device and 8F treated devices. Benefiting from the effective passivation and promoted carrier transport, the 8F CsPbI2Br device reveals the larger $R_{rec}$ than the Ref.

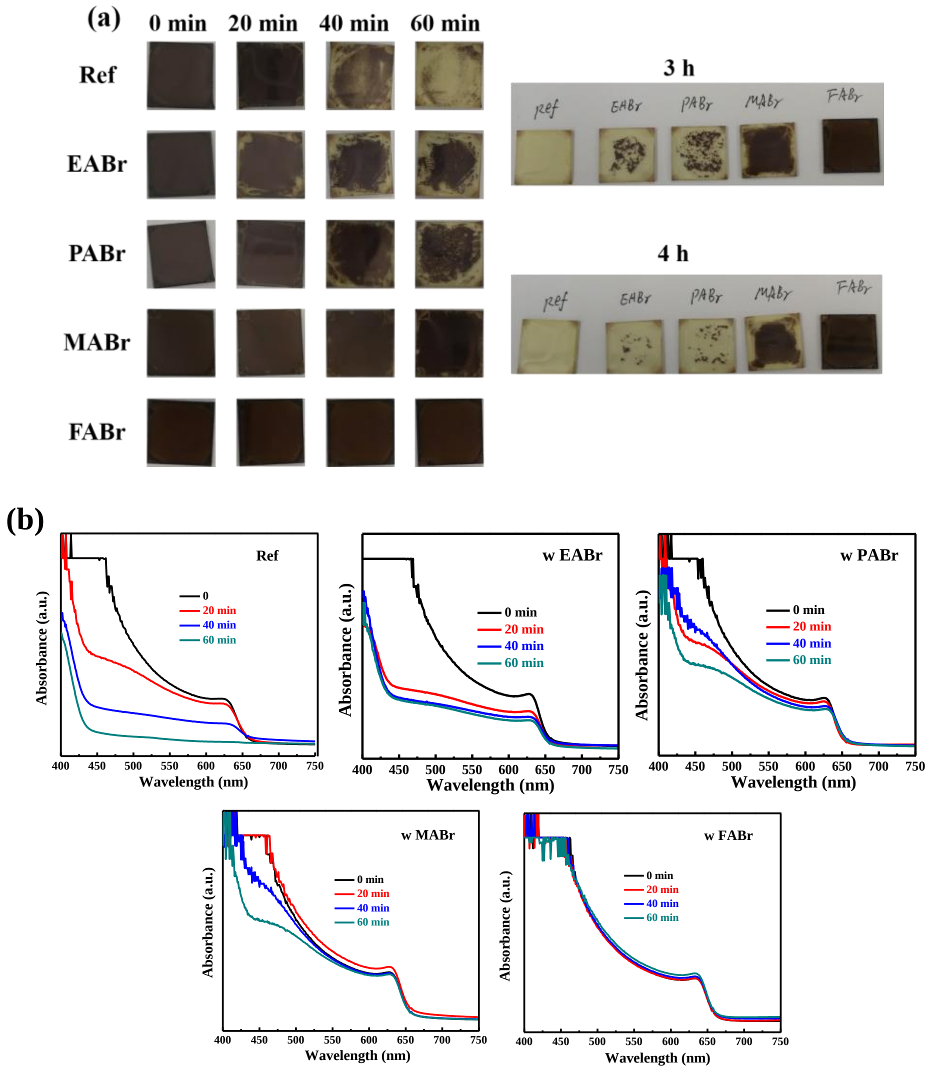


**Fig. S14** Phase stability of films treated with various ABr after exposing to an

ambient environment with controlled RH 40%. (**a**) Photograph of various films VS aging time. (**b**) Related absorption spectra. Based on these comparisons, it can conclude that the FABr treated filmis gifted the most tolerance from moisture erosion.

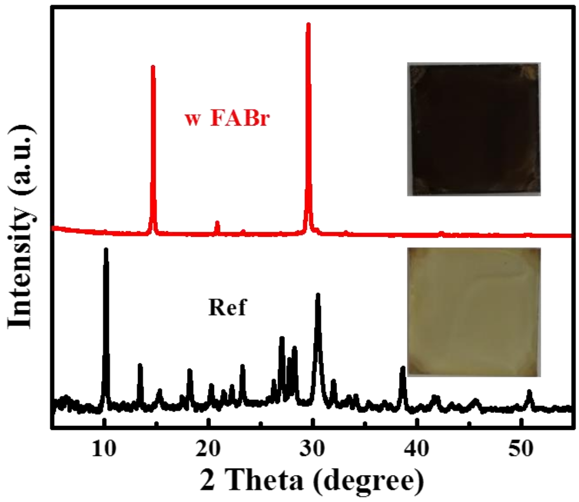


**Fig. S15** XRD patterns and photograph of the Ref and 8F treated film after aging under RH 40% for 5 h. The 8F film remains α phase (black film) while the Ref have transmitted into γ phase (yellow color film)

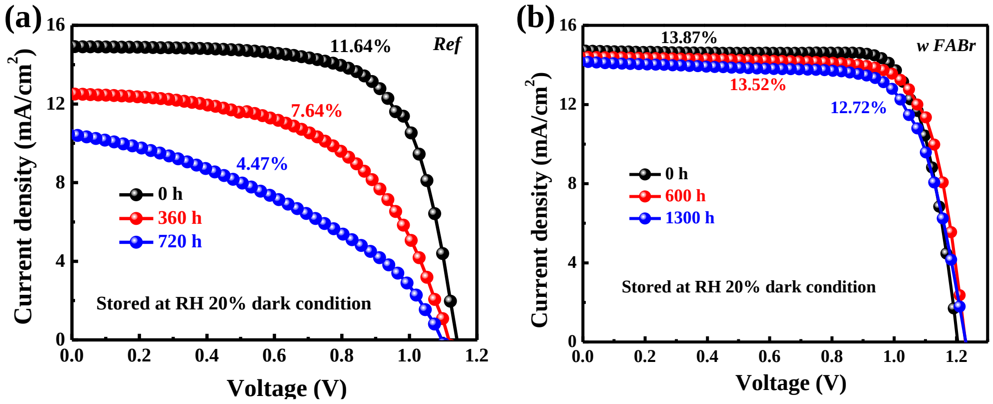


**Fig. S16** *J-V* curves of the devices aged under RH 20%: (**a**) Ref and (**b**) 8F devices

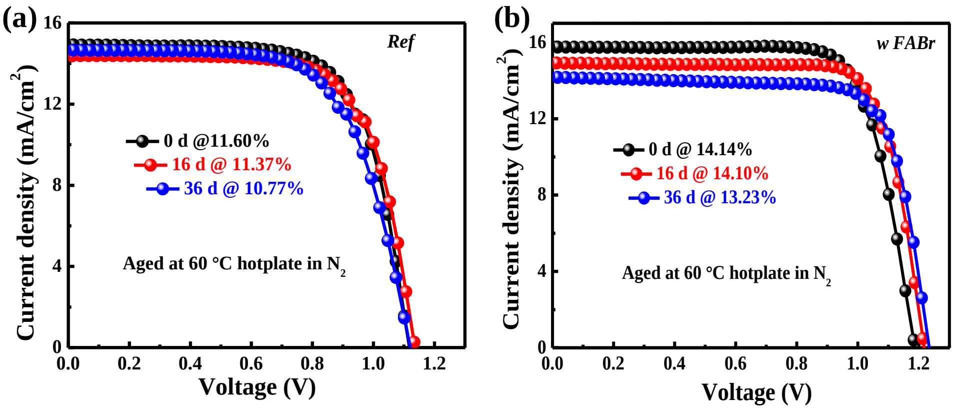


**Fig. S17** *J-V* curves of the devices aged at 60 °C hotplate: (**a**) Ref and (**b**) 8F devices

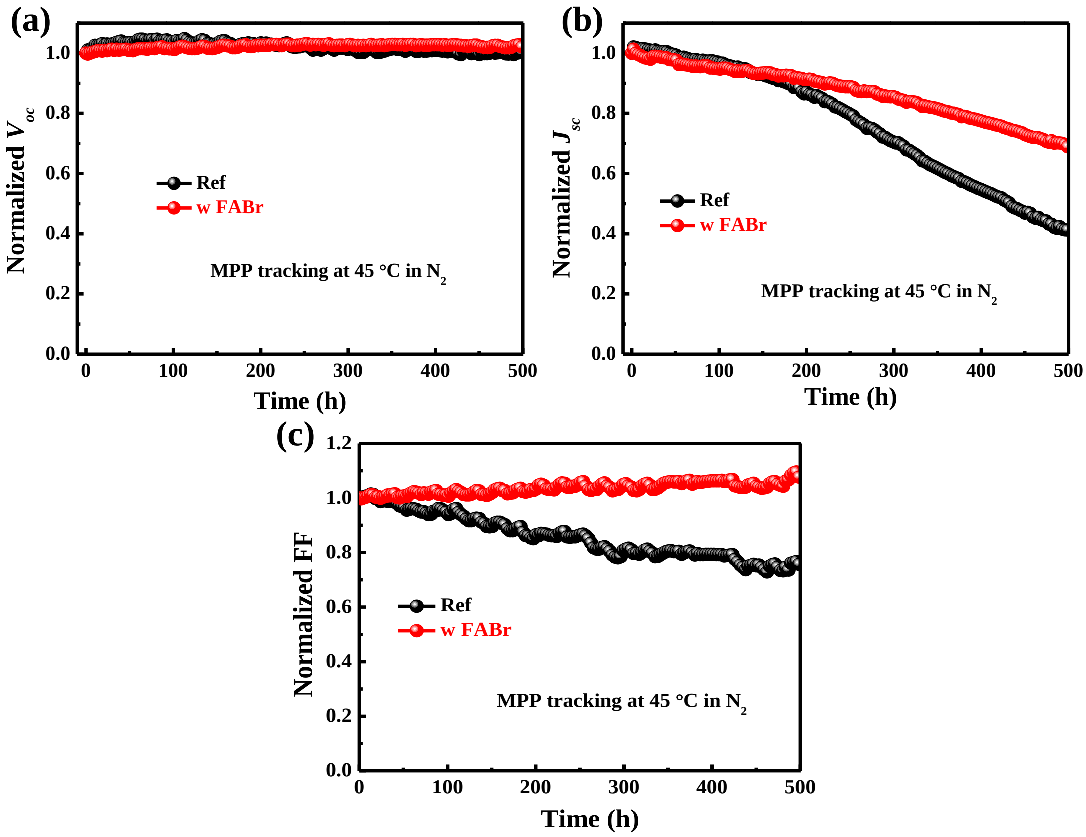


**Fig. S18** Photovoltaic parameters changes under MPP tracking at 45 °C for 500 h

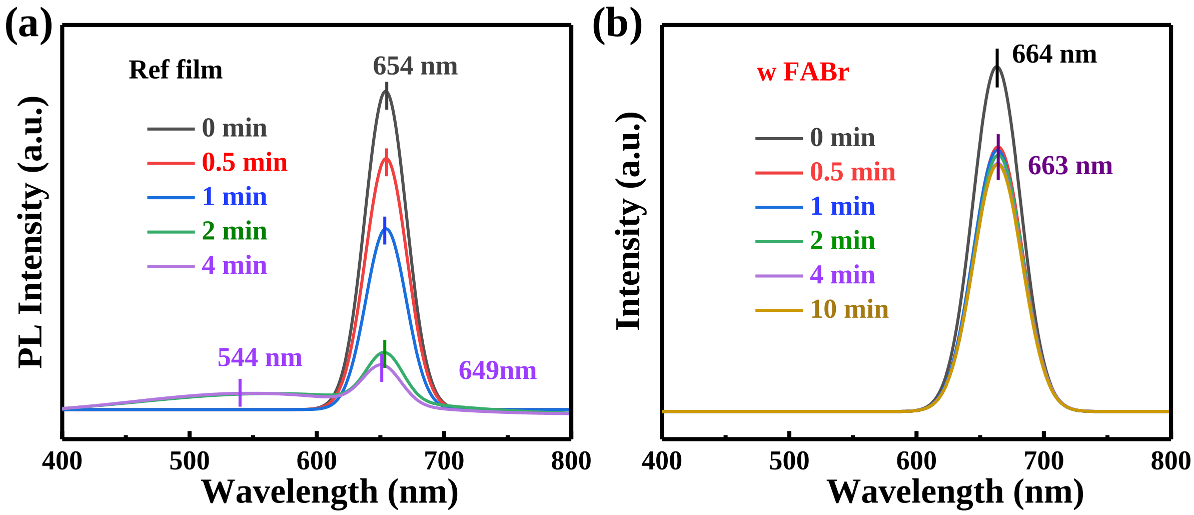


**Fig. S19** PL spectra of the Ref (**a**) and FABr (**b**) modified films illuminated with the 193 nm UV laser with energy of 3 mJ in vacuum box. The Ref film reveals the serious phase separation while the treated film with good stability [S32].

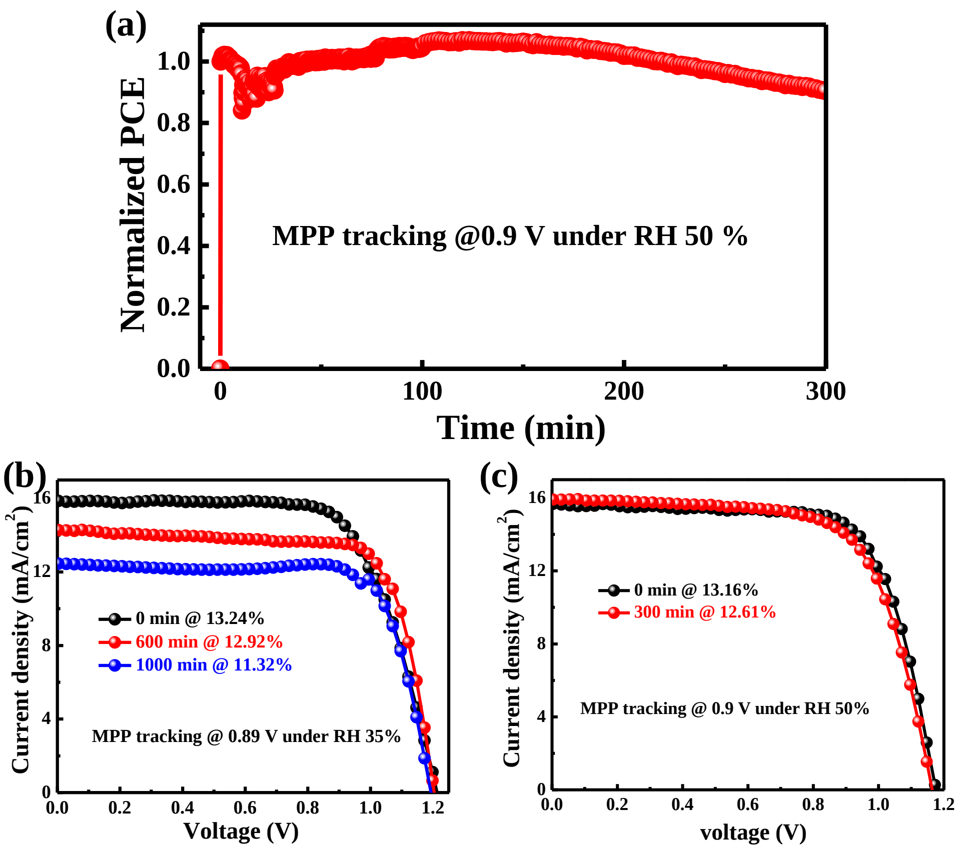


**Fig. S20** MPP measurements under ambient condition. (**a**) MPP plots of the device under RH 50% for 300 min. (**b**) *J-V* curves of the device after MPP tracking under RH 35%. (**c**) J-V curves of the device after MPP measurement under RH 50% for 300 min

## Supplementary References

[S1] R. E. Beal, D. J. Slotcavage, T. Leijtens, A. R. Bowring, R. A. Belisle et al., Cesium lead halide perovskites with improved stability for tandem solar Cells. J. Phys. Chem. Lett. **7,** 746 (2016). https://doi.org/10.1021/acs.jpclett.6b00002

[S2] S. Zhang, S. Wu, W. Chen, H. Zhu, Z. Xiong et al., Solvent engineering for efficient inverted perovskite solar cells based on inorganic $CsPbI_2Br$ light absorber. Mater. Today Energy **8**, 125 (2018). https://doi.org/10.1016/j.mtener.2018.03.006

[S3] H. Rao, S. Ye, F. Gu, Z. Zhao, Z. Liu, Z. Bian, C. Huang, Morphology controlling of all-inorganic perovskite at low temperature for efficient rigid and flexible solar cells. Adv. Energy Mater. **8**, 1800758 (2018). https://doi.org/10.1002/aenm.201800758

[S4] C. Liu, W. Li, C. Zhang, Y. Ma, J. Fan, Y. Mai, All-Inorganic $CsPbI_2Br$ perovskite solar cells with high efficiency exceeding 13%. J. Am. Chem. Soc.

**140**, 3825 (2018). https://doi.org/10.1021/jacs.7b13229

[S5] C. Liu, W. Li, H. Li, H. Wang, C. Zhang et al., Structurally reconstructed $CsPbI_2Br$ perovskite for highly stable and square-centimeter all-inorganic perovskite solar cells. Adv. Energy Mater. **9**, 1803572 (2019). https://doi.org/10.1002/aenm.201803572

[S6] S. Zhang, W. Chen, S. Wu, R. Chen, Y. Huang et al., A general strategy to prepare high-quality inorganic charge-transporting layers for efficient and stable all-layer-inorganic perovskite solar cells. J. Mater. Chem. A **7**, 18603 (2019). https://doi.org/10.1039/C9TA05802H

[S7] X. Liu, Y. Xiao, Q. Zeng, J. Jiang, Y. Li, Large-area organic-free perovskite solar cells with high thermal stability. J. Phys. Chem. Lett. **10**, 6382 (2019). https://doi.org/10.1021/acs.jpclett.9b02644

[S8] S. Zhang, W. Chen, S. Wu, R. Chen, Z. Liu et al., Hybrid inorganic electron-transporting layer coupled with a halogen-resistant electrode in $CsPbI_2Br$-based perovskite solar cells to achieve robust long-term stability. ACS Appl. Mater. Interfaces **11**, 43303 (2019). https://doi.org/10.1021/acsami.9b17464

[S9] L. Chen, L. Wan, X. Li, W. Zhang, S. Fu et al., Inverted all-inorganic $CsPbI_2Br$ perovskite solar cells with promoted efficiency and stability by nickel incorporation. Chem. Mater. **31**, 9032 (2019). https://doi.org/10.1021/acs.chemmater.9b03277

[S10] C. Liu, Y. Yang, C. Zhang, S. Wu, L. Wei et al., Tailoring C60 for efficient inorganic $CsPbI_2Br$ perovskite solar cells and modules. Adv. Mater. **32**, e1907361 (2020). https://doi.org/10.1002/adma.201907361

[S11] K. Jiang, J. Wang, F. Wu, Q. Xue, Q. Yao et al., Dopant-free organic hole-transporting material for efficient and stable inverted all-inorganic and hybrid perovskite solar cells. Adv. Mater. **32**, e1908011 (2020). https://doi.org/10.1002/adma.201908011

[S12] P. Wang, X. Zhang, Y. Zhou, Q. Jiang, Q. Ye et al., Solvent-controlled growth of inorganic perovskite films in dry environment for efficient and stable solar cells. Nat. Commun. **9**, 2225 (2018). https://doi.org/10.1038/s41467-018-04636-4

[S13] D. Bai, J. Zhang, Z. Jin, H. Bian, K. Wang et al., Interstitial $Mn^{2+}$-driven high-aspect-ratio grain growth for low-trap-density microcrystalline films for record efficiency $CsPbI_2Br$ solar cells. ACS Energy Lett. **3**, 970 (2018). https://doi.org/10.1021/acsenergylett.8b00270

[S14] H. Sun, J. Zhang, X. Gan, L. Yu, H. Yuan et al., Pb-reduced $CsPb_{0.9}Zn_{0.1}I_2Br$ thin films for efficient perovskite solar cells. Adv. Energy Mater. **9**, 1900896 (2019). https://doi.org/10.1002/aenm.201900896

[S15] K. L. Wang, R. Wang, Z. K. Wang, M. Li, Y. Zhang et al., Tailored phase transformation of $CsPbI_2Br$ films by copper(ii) bromide for high-performance all-inorganic perovskite solar cells. Nano Lett. **19**, 5176 (2019). https://doi.org/10.1021/acs.nanolett.9b01553

[S16] D. Bai, H. Bian, Z. Jin, H. Wang, L. Meng et al., Temperature-assisted crystallization for inorganic $CsPbI_2Br$ perovskite solar cells to attain high stabilized efficiency 14.81%. Nano Energy **52**, 408 (2018). https://doi.org/10.1016/j.nanoen.2018.08.012

[S17] L. Yan, Q. Xue, M. Liu, Z. Zhu, J. Tian et al., Interface engineering for all-inorganic $CsPbI_2Br$ perovskite solar cells with efficiency over 14%. Adv. Mater. **30**, e1802509 (2018). https://doi.org/10.1002/adma.201802509

[S18] W. Chen, H. Chen, G. Xu, R. Xue, S. Wang et al., Precise control of crystal growth for highly efficient $CsPbI_2Br$ perovskite solar cells. Joule **3**, 191 (2019). https://doi.org/10.1016/j.joule.2018.10.011

[S19] J. Tian, Q. Xue, X. Tang, Y. Chen, N. Li et al., Dual interfacial design for efficient $CsPbI_2Br$ perovskite solar cells with improved photostability. Adv. Mater. **31**, e1901152 (2019). https://doi.org/10.1002/adma.201901152

[S20] H. Zhao, Y. Han, Z. Xu, C. Duan, S. Yang et al., A novel anion doping for stable $CsPbI_2Br$ perovskite solar cells with an efficiency of 15.56% and an open circuit voltage of 1.30 V. Adv. Energy Mater. **9**, 1902279 (2019). https://doi.org/10.1002/aenm.201902279

[S21] W. Xu, F. He, M. Zhang, P. Nie, S. Zhang et al., Minimizing voltage loss in efficient all-inorganic $CsPbI_2Br$ perovskite solar cells through energy level alignment. ACS Energy Lett. **4**, 2491 (2019). https://doi.org/10.1021/acsenergylett.9b01662

[S22] Q. Xiao, J. Tian, Q. Xue, J. Wang, B. Xiong et al., Dopant-free squaraine-based polymeric hole-transporting materials with comprehensive passivation effects for efficient all-inorganic perovskite solar cells. Angew. Chem. Int. Ed. **58**, 17724 (2019). https://doi.org/10.1002/anie.201907331

[S23] Y. Zhang, C. Wu, D. Wang, Z. Zhang, X. Qi et al., High efficiency (16.37%) of cesium bromide—passivated all - inorganic $CsPbI_2Br$ perovskite solar cells. Solar RRL **3**, 1900254 (2019). https://doi.org/10.1002/solr.201900254

[S24] Y. Fan, J. Fang, X. Chang, M.-C. Tang, D. Barrit et al., Scalable ambient fabrication of high-performance $CsPbI_2Br$ solar cells. Joule **3**, 2485 (2019). https://doi.org/10.1016/j.joule.2019.07.015

[S25] W. Xiang, Z. Wang, D. J. Kubicki, W. Tress, J. Luo et al., Europium-doped $CsPbI_2Br$ for stable and highly efficient inorganic perovskite solar cells. Joule **3**, 205 (2019). https://doi.org/10.1016/j.joule.2018.10.008

[S26] J. Zhang, Z. Jin, L. Liang, H. Wang, D. Bai et al., Iodine-optimized interface

for inorganic $CsPbI_2Br$ perovskite solar cell to attain high stabilized efficiency exceeding 14%. Adv. Sci. **5**, 1801123 (2018). https://doi.org/10.1002/advs.201801123

[S27] E.C. Shen, J.D. Chen, Y. Tian, Y. X. Luo, Y. Shen et al., Interfacial energy level tuning for efficient and thermostable $CsPbI_2Br$ perovskite solar cells. Adv. Sci. **7**, 1901952 (2019). https://doi.org/10.1002/advs.201901952

[S28] S.C. Liu, Z. Li, Y. Yang, X. Wang, Y.X. Chen et al., Investigation of oxygen passivation for high-performance all-inorganic perovskite solar cells. J. Am. Chem. Soc. **141**, 18075 (2019). https://doi.org/10.1021/jacs.9b07182

[S29] J. Xue, R. Wang, K.L. Wang, Z.K. Wang, I. Yavuz et al., Crystalline liquid-like behavior: surface-induced secondary grain growth of photovoltaic perovskite thin film. J. Am. Chem. Soc. **141**, 13948 (2019). https://doi.org/10.1021/jacs.9b06940

[S30] S. Fu, W. Zhang, X. Li, L. Wan, Y. Wu et al., Dual-protection strategy for high-efficiency and stable $CsPbI_2Br$ inorganic perovskite solar cells. ACS Energy Lett. **5**, 676 (2020). https://doi.org/10.1021/acsenergylett.9b02716

[S31] Y. Han, H. Zhao, C. Duan, S. Yang, Z. Yang et al., Controlled n−Doping in Air-Stable $CsPbI_2Br$ Perovskite Solar Cells with a Record Efficiency of 16.79%. Adv. Funct. Mater. **30**, 1909972 (2020). https://doi.org/10.1002/adfm.201909972

[S32] H. Zhang, X. Fu, Y. Tang, H. Wang, C. Zhang et al., Phase segregation due to ion migration in all-inorganic mixed-halide perovskite nanocrystals. Nat. Commun. **10**, 1088 (2019). https://doi.org/10.1038/s41467-019-09047-7
